# Clinical Trials Focusing on Drug Control and Prevention of Ventilator-Associated Pneumonia: A Comprehensive Analysis of Trials Registered on ClinicalTrials.gov

**DOI:** 10.3389/fphar.2018.01574

**Published:** 2019-02-26

**Authors:** Lingmin Chen, Yanling Su, Liuliu Quan, Yonggang Zhang, Liang Du

**Affiliations:** ^1^Department of Anesthesiology and National Clinical Research Center for Geriatrics, West China Hospital, Sichuan University, Chengdu, China; ^2^West China School of Medicine, Sichuan University, Chengdu, China; ^3^Unit of General Practice, West China Hospital, Sichuan University, Chengdu, China; ^4^Department of Periodical Press and National Clinical Research Center for Geriatrics, West China Hospital, Sichuan University, Chengdu, China; ^5^Chinese Evidence-Based Medicine Center, West China Hospital, Sichuan University, Chengdu, China

**Keywords:** ventilator associated pneumonia, ClinicalTrials.gov, drug control, drug prevention, interventional clinical trials

## Abstract

**Objective:** Clinical trials have emerged as the main force in driving the development of medicine. However, little is known about the current status of clinical trials regarding drug control and prevention of ventilator-associated pneumonia (VAP). This study aimed at providing a comprehensive landscape of these trials on the basis of ClinicalTrials.gov.

**Methods:** A cross-sectional, descriptive study of clinical trials on drug control and prevention of VAP which have been registered on the ClinicalTrials.gov up to 25^th^ August 2018 was conducted.

**Results:** A total of 109 eligible trials were identified. Trials were started from 1998 to 2018, and most trials focused on adult patients. More than half trials were completed, while only 11.9% trials had results available. Sample sizes were relatively large, with a median enrollment of 146. Universities were listed as the primary sponsor for 36.7% trials, industry for 28.4% trials and hospitals for 19.3% trials. Of the 109 VAP trials, 37 trials were from in Europe, 36 in North America and 27 in Asia. Among the 97 interventional trials, 32 were phase 3 trials, 21 were phase 4 trials, and 16 were phase 2 trials. Most interventional trials were randomized trials with a parallel assignment, and 57.7% trials were blinded. Of the 12 observational trials, 9 were cohort studies, and 10 trials were prosepctive studies. Drugs about oral care, preemptive antibiotics and probiotics were most investigated for prevention. A total of 61 trials investigated drugs for the treatment of VAP, mainly focused on antibiotics. A total of 36 kinds of antibiotics were investigated for monotherapy or combination therapy. Beta-lactams were most studied, followed by aminoglycosides and polypeptides.

**Conclusion:** Most clinical trials registered on ClinicalTrials.gov about drugs for VAP were interventional trials with the purpose for treatment. A high proportion of interventional trials were randomized, parallel assigned and masked. Our analysis highlights the need for improvement in completeness of study results on the ClinicalTrials.gov.

## Introduction

Ventilator-associated pneumonia (VAP) is defined as nosocomial pneumonia that occurs in mechanically ventilated patients at least 48 h after the initiation of mechanical ventilation (Bassetti et al., 2012). Although the incidence of VAP varies according to the population and the different criteria used, VAP remains the leading cause of nosocomial infection in the intensive care unit (Ego et al., 2015). VAP has been associated with worsened patient outcomes, increased hospitalization cost and higher mortality rates (Roberts et al., 2017). A number of evidence-based strategies have been used for the prevention and treatment of VAP (Davis, 2006; Liapikou et al., 2014). The primary treatment is the use of antimicrobial agents. With the increase of multidrug-resistant infections, it becomes more difficult to select appropriate antibiotics for patients with VAP (Roberts et al., 2017). More efforts are needed to develop more effective prevention and treatment strategies.

Clinical trials are the most effective ways for evaluating preventive and therapeutic strategies (Feizabadi et al., 2017; Ranawaka et al., 2018). ClinicalTrials.gov is a public trial registry provided by the U.S. National Library of Medicine and the U.S. Food and Drug Administration. In 2005, all International Committee of Medical Journal Editors (ICMJE) member journals released a statement that clinical trials must be publicly registered in trials’ registries before they were considered for publication (De Angelis et al., 2004). ClinicalTrials.gov registry which contains over 289,000 research studies conducted in 205 countries, is supposed to be the most comprehensive source for ongoing or completed clinical trials (Califf et al., 2012; Goswami et al., 2013; Bell and Tudur Smith, 2014). Evaluating this registered clinical trials will enable us to gain a deeper understanding of drug control and prevention of VAP. In this study, we aimed to examine the characteristics of registered clinical trials regarding drug control and prevention of VAP.

## Materials and Methods

A cross-sectional, descriptive study of clinical trials on drug control and prevention of VAP which had been registered on the ClinicalTrials.gov database was conducted. The trials were obtained from ClinicalTrials.gov using the advanced search function with the search term “ventilator-associated pneumonia” for “condition” on August 25^th^, 2018. All of these searched clinical trials were gone through to identified records of all pharmaceutical trials. We extracted all the following information: registered number, study type, start date, the status of trial, study result, study sample, participant age, primary sponsor, location, primary purpose, the phases of trial, allocation, intervention model, masking and intervention. All trials were then further subclassified according to study type. We used descriptive statistics to characterize trial categories. Frequencies and percentages were provided for categorical data. Medians and interquartile ranges were used for continuous variables.

## Results

The initial search found 232 clinical trials registered on ClinicalTrials.gov up to August 25^th^, 2018. After carefully reviewing all the information, 123 trials were excluded, including 10 trials not about VAP and 113 not pharmaceutical trials in patients with VAP). Thus, a total of 109 registered trials were included.

### Basic Characteristics of Included Trials

Basic characteristics of the identified trials are shown in Table 1. Among the 109 eligible trials, 97 (89.0%) were interventional trials, and the 12 (11.0%) were observational trials. The trials were started from 1998 to 2018, with maximum studies started from 2012 to 2016. The status of more than half trials were completed (*n* = 59, 54.1%), followed by recruiting (*n* = 15, 13.8%). While 13 trials were terminated and 2 trials were withdrawn. Only 11.9% trials had results available, and 24.7% trials had web links for the publications of results on the ClinicalTrials.gov. Trials about VAP were mainly focused on adult patients, with 88.1% trials included only adult patients. Samples sizes were relatively large, with 62.9% trials enrolling 100 or more participants. The median number of participants per trial was 146 (66–270). Universities were listed as the primary sponsor for 40 (36.7%) trials, hospitals for 21 (19.3%) trials and industries for 31 (28.4%) trials. Of the 109 VAP trials, 37 (33.9%) trials were conducted in Europe, 36 (33.0%) in North America and 27 (24.8%) in Asia. 25.7% of trials were supported by industry funding.

**Table 1:** Characteristics of included trials.

Characteristics		Number	Percentage (%)
Study type			
	Interventional	97	89.0
	Observational	12	11.0
Year			
	Prior to 2006	23	21.1
	2006–2009	20	18.3
	2009–2012	15	13.8
	2012–2016	31	28.4
	2016–2018	20	18.3
Status			
	Completed	59	54.1
	Recruiting	15	13.8
	Active, not recruiting	1	0.9
	Not yet recruiting	4	3.7
	Enrolling by invitation	1	0.9
	Withdrawn	2	1.8
	Terminated	13	11.9
	Unknown status	14	12.8
Study results			
	No available results	96	88.1
	Has available results	13	11.9
Participant age (y)			
	<18	7	6.4
	> = 18	96	88.1
	All	6	5.5
Enrollment			0.0
	< = 50	24	22.0
	50–100	17	15.6
	100–500	52	48.1
	> = 500	16	14.8
Sponsor			
	University	40	36.7
	Hospital	21	19.3
	Industry	31	28.4
	Other	17	15.6
Location			
	Europe	37	33.9
	North America	36	33.0
	Asia	27	24.8
	South America	6	5.5
	Oceanic	2	1.8
	Africa	1	0.9
Funded by			
	Other	62	56.9
	Industry	28	25.7
	Other and Industry	15	13.8
	Other and NIH	3	2.8
	Other and U.S. Fed	1	0.9


### Characteristics of Study Design

Study design characteristics of interventional trials are displayed in Table 2. Of the 97 interventional trials, 32 (33.0%) were phase 3 trials, 21 (21.6%) were phase 4 trials, and 16 (16.5%) were phase 2 trials. Primary purposes of interventional trials were the treatment (63.9%) and prevention (33.0%). Most trials were randomized trials with the parallel assignment. Among the 97 interventional trials, 41 (42.3%) trials were without masking, 21 (21.6%) with quadruple masking, 15 (15.5%) with double masking, 12 (12.4%) with triple masking and 9 (9.3%) with single masking. Study design characteristics of observational trials are shown in Table 3. Of the 12 interventional trials, 9 (75.0%) were cohort studies, and 1 (8.3%) was a case-only study. Ten (83.3%) trials were prospective and 2 (16.7%) were retrospective.

**Table 2:** Study design elements of interventional trials.

Characteristics		Number	Percentage (%)
Primary purpose			
	Treatment	62	63.9
	Prevention	32	33.0
	Other	3	3.1
Phases			
	Phase 1	6	6.2
	Phases 1/2	2	2.1
	Phase 2	16	16.5
	Phases 2/3	6	6.2
	Phase 3	32	33.0
	Phase 4	21	21.6
	Not applicable	14	14.4
Allocation			
	Randomized	88	90.7
	Non-randomized	3	3.1
	Missing value	6	6.2
Intervention model			
	Parallel assignment	83	85.6
	Factorial assignment	2	2.1
	Crossover assignment	3	3.1
	Single group assignment	9	9.3
Masking			
	Single	9	9.3
	Double	15	15.5
	Triple	12	12.4
	Quadruple	21	21.6
	None	41	42.3


**Table 3:** Study design elements of observational trials.

Characteristics		Number	Percentage (%)
Model			
	Case-only	1	8.3
	Cohort	9	75.0
	Missing value	2	16.7
Time perspective			
	Prospective	10	83.3
	Retrospective	2	16.7


### Overview of Investigated Drugs

A total of 32 trials investigated the drugs for the prevention of VAP, mainly focused on oral care, preemptive antibiotics and probiotics. A summary of studied drugs for prevention is provided in Table 4. Most of the trials investigated the effect of chlorhexidine (*n* = 13) for oral care. Nine trials investigated preemptive antibiotics, and five studies investigated the effect of probiotics in the prophylaxis of VAP. Sixty nine trials investigated the drugs for the treatment of VAP, mainly focused on antibiotics. An overview of drugs for the treatment of VAP is shown in Table 5. A total of 36 kinds of antibiotics were investigated for monotherapy or combination therapy. Beta-lactams were most studied, followed by aminoglycosides and polypeptides. Doripenem (*n* = 7), colistin (*n* = 7), and vancomycin (*n* = 6) were the three most investigated drugs. Thirteen trials investigated the effect of aerosolized antibiotics, including colistin, amikacin, ceftazidime, tobramycin, ancomycin, and gentamicin and five trials investigated the duration of antibiotic treatment. Other treatments involving oral care, stress ulcer prophylaxis and simvastatin therapy were also investigated.

**Table 4:** Overview of drugs for prevention.

Types	Name and number of investigated drug
Oral care	Chlorhexidine (13)
	Potassium permanganate (1), Povidone iodine (1)
Antibiotic	Amikacin (1), ASN100 (1), Azithromycin (1), Ceftriaxone (1), Colistin (1), KB001 (1), Iseganan hydrochloride (1), Sultamicillin (1), Neomycin/Colistin/Nystatin/Vancomycin (1)
Others	*Lactobacillus* (5)
	Insulin (1), Atorvastatin (1), Pravastatin (1), Atropin (1)


**Table 5:** Overview of drugs for treatment.

Types		Name and number of investigated drug
Antibiotic	Beta-lactams	Doripenem (7)
		Ceftobiprole (4)
		Meropenem (3)
		Tazobactam (2)
		Imipenem (2)
		Piperacillin/Tazobactam (2)
		Ceftolozane/Tazobactam (2)
		Cefiderocol(S-649266)(2)
		Amoxicillin (1), Ceftazidime (1), Cefepime (1), Piperacillin (1), Cilastatin (1), Meropenem (1), Relebactam (MK-7655) (1), Amoxicillin/Clavulanate potassium (1), Aztreonam/Avibactam (1), Ceftazidime/Avibactam (1), Imipenem/Relebactam (1), Imipenem/Relebactam (1)
	Aminoglycosides	Amikacin (3)
		Gentamicin (2)
		Tobramycin (2)
	Polypeptides	Colistin (7)
		Vancomycin (6)
		Murepavadin (POL7080) (2)
		Polymyxin B (1), Colimycin (1)
	Others	Linezolid (5)
		Quinolone (1), Clarithromycin (1), Iclaprim (1), Metronidazole (1), Cotrimoxazole (1)
	Beta-lactams/aminoglycosides	Ceftazidime/Amikacin (1), Imipenem/Amikacin (1), Augmentin/Ceftriaxone/Cefotaxime/Netilmycin/Tobramycin (1)
	Beta-lactams/polypeptides	Imipenem/Cilastatin (3)
		Colistin/Imipenem (1), Polymyxin B/Doripenem (1)
	Beta-lactams/others	Linezolid/Ceftazidime (3)
	Others/others	Linezolid/Vancomycin (1)
Others		*Lactobacillus* (2)
		Chlorhexidine (1), Ranitidine (1), Sucralfate (1), Simvastatin (1), IC43 (1), KBPA-101 (1)


## Discussion

This study provides a descriptive assessment of trials registered on ClinicalTrials.gov about drug control and prevention of VAP. We found the vast majority were interventional trials. More than half trials were completed, however, only 11.9% trials had available results. Relatively high percentage (13.7%) of trials were terminated or withdrawn. Most trials registered were relatively large samples, with the median number of 146 participants per trial. We demonstrated that interventional VAP trials were predominantly late-phase studies with a generally high proportion of randomized and parallel assignment studies. Drugs about oral care, preemptive antibiotics and probiotics were investigated for prevention. Drugs for treatment were mainly focused on antibiotics, with beta-lactams being most studied.

It is reassuring that most interventional studies were randomized (90.7%), parallel assigned (85.6%), and masked (57.7%). A further 21.6% of registered trials were quadruple masking. These values are higher than which have been reported among interventional clinical trials registered in the ClinicalTrials.gov database during 2007 to 2010 (Califf et al., 2012). Randomization is regarded as a hallmark of a high-quality clinical trial (Moher, 1998). Randomization and blinding can reduce bias and make evidence more reliable. It is important for researchers to adopt randomization and blinding when feasible. Owing to the limited information on ClinicalTrials.gov, we could not judge whether all the trials were appropriately designed. Research in children are often regarded as challenging due to scientific, ethical and practical difficulties (Steinbrook, 2002; Pasquali et al., 2012). All the interventional trials registered in ClinicalTrials.gov from 2007 to 2010, only 17% trials included children (Califf et al., 2012). Trials about VAP were also mainly focused on adult patients, only 11.9% trials included children. More work is needed to ensure that children are involved in clinical trials of drugs so that more information could be provided for prescribing medications in children.

More than half trials were completed, however, only 11.9% trials had results available on the ClinicalTrials.gov, and 24.7% trials had web links for publications of results on the ClinicalTrials.gov. Similar non-publication rates of completed studies have been reported for clinical trials on the ClinicalTrials.gov (Riveros et al., 2013; Roberto et al., 2018). Evidence indicates that positive trials were submitted more rapidly after completion and were published more rapidly after submission (Ioannidis, 1998). Studies sponsored by pharmaceutical companies are unlikely to publish negative results or reports not favoring the funding entities (Lexchin et al., 2003; Bourgeois et al., 2010). Consequently, there could be a potential for publication bias. The ClinicalTrials.gov registry was created to make research more transparent and reduce publication bias. Publication of study results should be encouraged for all trials. Efforts should be made to let results of all completed studies available in the ClinicaTrials.gov registry in no delay. The percentage of terminated or withdrawn trials in our study was relatively high (13.7%). Califf et al. (2012) found that the rate of interventional trials registered between 2007 and 2010 that was terminated or withdrawn trials was only 3.3% (Califf et al., 2012). We further explored and found that financial issues and lack of enrollment were the main reasons. Besides, one trial was stopped for futility after first scheduled analysis of recruitment of 300 participants (Papazian et al., 2013). One trial was stopped after a scheduled review of data showing lower cure rates and higher mortality rates in one of the treatment groups (Kollef et al., 2012).

Compared with trials for prevention, more trials were treatment-oriented, mainly focused on antibiotics. The right choice of the antimicrobial treatment is key to the prognosis of patients with VAP (Craven et al., 2014). The increase of multi-drug resistant organisms in HAP/VAP, especially gram-negative bacilli, make the selection of effective drugs more difficult (Falco et al., 2016). A total of 36 kinds of antibiotics were investigated for the treatment of VAP. However, most trials evaluated previously approved antibiotics, novel antimicrobial agents were less evaluated. Novel drugs for the treatment of VAP could improve the therapeutic options available and may offer a potential new opportunity to patients who have multi-drug resistant organisms. Thirteen studies investigated nebulized antibiotics for the treatment for VAP. Aerosolized antibiotics delivered through mechanical ventilation can achieve higher airway-drug concentrations with reduced systemic toxicity (Vincent et al., 2016). There could be a place for nebulized antibiotics in VAP.

Our study had several limitations. First, ClinicalTrials.gov does not include all clinical trials. However, as ClinicalTrials.gov accounts for more than 80% of all studies in the World Health Organization’s International Clinical Trials Registry Platform (Zarin et al., 2007). So our analysis is broadly representative. Second, our study provides only an overview of the characteristics of the registered trials. The actual strengths and weaknesses of the clinical studies were not assessed. The validity of the data in ClinicalTrials.gov is dependent on the quality of information provided by the sponsors. The missing data in some fields may introduce some bias into the results. Thirdly, we did not examine whether the registered trials have yet published the data in peer-reviewed journals. The proportion of trials that was published in the peer-reviewed literature requires further study.

The current study provides an overview of registered trials about drug control and prevention of VAP. It appears that most registered clinical trials are interventional trials with the purpose for treatment. Interventional trials tend to have a good trial design, with a relatively high proportion of randomized, blind and parallel assignment studies. Our analysis highlights the need for improvement in completeness of study results on the ClinicalTrials.gov.

## Author Contributions

YZ and LD provided the source for the study and edited the manuscript. LC and YS searched the data, extracted the data, assessed the data, analyzed the data, and drafted the manuscript. LQ analyzed the data.

## Conflict of Interest Statement

The authors declare that the research was conducted in the absence of any commercial or financial relationships that could be construed as a potential conflict of interest.
